# C-71980262, a novel small molecule against human papilloma virus-16 E6 (HPV-16 E6) with anticancer potency against cervical cancer: A computational guided *in vitro* approach

**DOI:** 10.22038/ijbms.2024.78090.16882

**Published:** 2024

**Authors:** Ashish Kumar

**Affiliations:** 1 Department of Microbiology & Clinical Parasitology, College of Medicine, King Khalid University, Abha, Saudi Arabia

**Keywords:** Apoptosis, Cervical cancer, E6 High-throughput virtual- screening, HPV, Molecular dynamic-simulations, p53

## Abstract

**Objective(s):**

Human papillomavirus-16 E6 (HPV-16 E6) forms a heterodimer complex to up-regulate the degradation of tumor suppressor protein p53 to promote cervical cancer. This study aims to identify a novel small molecule against E6 with anticancer efficacy against HPV-16, a prime high-risk serotype inducer for cervical cancer.

**Materials and Methods:**

Autodock-vina-based high-throughput virtual screening and atomistic molecular dynamic simulations were used for identification of targeted lead molecules. HPV-16 infected SiHa and CaSki cell lines were used to validate the lead compound in vitro. Proliferation of cancer cells was analyzed by MTT assay and flow cytometry was used to analyze target inhibition, apoptosis, and p53.

**Results:**

High throughput virtual screening and molecular dynamic simulation identified C-71980262 as a lead candidate that could bind HPV-E6. Atomistic molecular dynamic simulation of E6 bound C-71980262 for 200 ns showed that the predicted ligand binding was stable with minimal energy expenditure, proposing the viability and veracity of the assessed molecule. C-71980262 inhibited the proliferation of SiHa and CaSki cells with GI50 values of 355.70 nM and 505.90 nM, respectively. The compound reduced HPV-16 E6 while inducing early and late-phase apoptosis in these cells. Treatment with C-71980262 increased the p53-positive populations in SiHa and CaSki cells.

**Conclusion:**

C-71980262 was identified as a novel lead molecule that could inhibit the HPV-16 E6 and increase p53 in cervical cancer cells. Further in vitro and in vivo validation is warranted to consolidate and corroborate this lead compound against HPV-induced cancer progression.

## Introduction

Human papillomavirus (HPV) is thought to be responsible for more than 90% of cervical cancers (1). The link between genital HPV infection and cervical cancer was first established by the German scientist Harold Zur Hausen in 1980 (2). Since then, scientists have identified 30 types of HPV infections that are transmitted through sexual contact. These HPVs are part of the *Papillomaviridae* family, characterized as small, non-enveloped, double-stranded, circular DNA viruses with an infectious capsid, capable of targeting epithelial cells in genital mucosa (3, 4). Mucosal HPV infections are spread primarily through sexual intercourse, with a preference for the junction between the endocervix and ectocervix as the initial site of infection (5). HPV is categorized into five genera viz., alpha (65 types), beta (53 types), gamma (98 types), mu (3 types), and nu (1 type)(6). Depending on the body region they infect, HPVs can be further classified as cutaneous or mucosal types. Among these types, subcategorization into low-risk (LR) and high-risk (HR) groups is based on their association with cervical cancer and precursor lesions (5, 7). Notably, HPV-16 and HPV-18 of the alpha genus, both high-risk HPV, are responsible for causing approximately 80% of cervical cancer cases (8). 

The HPV genome is divided into three distinct regions: an early region with seven proteins (E1-7), a late region with two proteins (L1-2), and an upstream regulatory region (URR)(9, 10). HPV-16, a significant contributor to cervical cancer, relies on Normal Immortal Keratinocytes (NIK) or Human Foreskin Keratinocytes (HFK) with undying telomerase activity to support high-risk alpha HPV (HPV-16) by recruiting two oncoproteins, E6 and E7 (11, 12). HPV-16 lacks a reproductive system and instead exploits the host’s epithelium cell division mechanism, taking control of the cell cycle and apoptotic processes to facilitate viral replication. 

The oncogenesis driven by HPV is primarily the result of two cooperative oncogenes, E6 and E7, responsible for viral replication and tumorigenesis. HPV-16, a critical high-risk HPV, has an oncoprotein E6 consisting of 158 amino acids with N-terminal and C-terminal domains, as well as two Zinc-finger domains. These zinc-finger domains, composed of pairs of Cys-X2-Cys-X29-Cys-X2-Cys motifs, create a deep pocket essential for binding to E6AP (E6-associated protein)(13, 14). These motifs are highly conserved in E6 proteins, maintaining their purity to enable the protein’s oncogenic functions. The carboxy-terminal domain of E6 includes a PDZ-binding motif, which interacts with cellular proteins such as p53, leading to its degradation (14, 15). HPV-16 E6 plays a pivotal role in the viral life cycle and various oncogenic processes, including uncontrolled cell division, cell survival and proliferation, differentiation, angiogenesis, invasion, metabolic reprogramming, metastasis, and anti-apoptotic effects. E6 is a biomarker in cervical cancer patients, and collectively, E6 and E7 are referred to as “oncoplayers.” E6 modulates multiple cellular processes through protein-protein interactions, disrupting the tumor-inhibiting quality of p53 (16, 17). 

The tumor suppressor p53, often referred to as the “guardian of the genome,” is degraded through ubiquitination due to the formation of a trimeric ternary complex of E6-p53-E6AP (18). In normal scenarios, p53 is regulated by murine double minute 2 (Mdm2), a RING finger domain-containing ubiquitin ligase. However, during viral infection, p53 is phosphorylated, leading to the inactivation of the Mdm2 pathway and causing p53 transcriptional failure. Consequently, E6 can repress p53 through both proteasomal and non-proteasomal mechanisms. While p53 is the primary target of E6, its anti-apoptotic activity also involves targeting other pro-apoptotic agents like Bak and Bax, which are vital for the mitochondria-dependent intrinsic apoptotic pathways (19). E6 targets Bak for proteasome-mediated degradation via E6AP, leading to anti-apoptosis. E6 also disrupts other cellular processes, such as regulation of c-myc, which contributes to the immortality of cancer cells. It mediates host apoptotic systems like tumor necrosis factor receptor 1 (TNFR1) and Fas-associated death domain to accelerate degradation (20-22). Additionally, E6 influences the telomere machinery by activating telomerase and preventing telomere shortening, thus promoting continuous cell division through the transcriptional activation of the telomerase catalytic subunit hTERT (human Telomerase reverse transcriptase). The activation of hTERT by E6 is crucial in the development of malignant cervical cancer. E6 also down-regulates NFϰB, which may play a role in cytokine production for the immune response in cancer, albeit with varying functions in different stages of viral infection (23).

Intrinsically and extrinsically, the pathways for apoptosis are essential in maintaining a balance between pro-apoptotic and anti-apoptotic proteins. However, the presence of oncogenes can disrupt these pathways. Silencing the critical oncogene E6 using small molecules leads to an increase in p53 levels, activating the PUMA (p53 up-regulated modulator of apoptosis) to promote and direct the cell toward apoptosis. To achieve this therapeutic outcome, effective targeting of E6 is crucial, requiring a comprehensive understanding of the druggable pockets in E6 and their interactions with protein molecules. Previous structural knowledge of E6 shows the existence of a hydrophobic site that interacts with p53 and E6AP. Targeting this area can prevent the formation of the trimeric complex and provides a framework for designing suitable drugs to inhibit these interactions, thereby counteracting oncogenesis.

## Materials and Methods

***Materials***

All reagents and chemicals used in this study were obtained from Sigma Aldrich (St. Louis, MO, USA). Non-cancer Vero and HPV-16 positive human cervical carcinoma cells (SiHa and CaSki cells) were obtained from ATCC (ATCC, Rockville, MD, USA) and cultured according to the supplier’s instructions. The lead compound from ChemBridge was dissolved in DMSO stored at -20 °C. The Annexin V assay kit was from bioscience- ThermoFisher Scientific (Waltham, MA, USA). FITC coated anti HPV-16 E6 antibody was from Santa Cruz Biotechnology (Texas, MA, USA). FITC tagged anti p53 antibody was from ThermoFisher Scientific (Waltham, MA, USA).

***Methods***

***Structure retrieval and high-throughput virtual screening***

The crystal structure of E6-HPV16 was retrieved from the PDB database (6SIV) and subjected to preprocessing using Discovery Studio Visualizer. This involved the removal of crystal waters and addition of polar hydrogens. The SwissPDB viewer was utilized to address missing side chains and atoms. A docking grid box, based on a protein-protein interaction interface, was generated with dimensions of 20 units on all sides. High-throughput virtual screening of the preloaded ChemBridge library, limited to molecules with a molecular weight (MW) between 150 to 350 Da, was conducted using the D-HTVS module from SiBioLEAD (24). The analysis of protein-ligand interactions was performed using the PLIP analysis plugin and the results were visualized through Discovery Studio Visualizer.

***Molecular dynamic simulation***

The Molecular Dynamics (MD) simulation of E6-HPV16 in complex with the target ligand was computed through an MD simulation server that utilizes the GROMACS simulation package, accessible at www.sibioled.com. The ligand topology was generated using AMBERTOOLS and the ACPYPE package, and the simulation system was parameterized with the OPLS/AA force field(25). Initially, the protein-ligand complex was immersed in a triclinic box containing Simple Point Charge (SPC) water for hydration. To mimic physiological conditions, the simulation system was neutralized using NaCl as counterions, supplemented with an additional 0.15M NaCl salt. 

The simulation system, encompassing the protein-ligand complex, SPC water, and salt ions, underwent energy minimization for 5000 steps using the steepest descent method. The simulation system was equilibrated for 300 ps under constant temperature and pressure, using NVT/NPT protocol. The MD run extended for 200 ns utilizing a leap-frog integrator, capturing trajectory frames at every 20 ps. GROMACS in-built trajectory analysis scripts were employed for the analysis of simulation trajectories, and the results were visualized using xmgrace.

The Gibbs binding free energy (ΔG binding) of the protein-ligand complexes was computed employing the GMX_MMPBSA method(26, 27). Gibbs binding free energies were generated using trajectory frames saved at every 4 ns over the course of 200 ns.

***Cell proliferation inhibition assay***

Vero, SiHa, and CaSki cells were treated in a 24-well plate and treated with different concentrations of C-71980262 and incubated for 48 hr. Cell proliferation inhibition was analyzed using MTT assay as reported elsewhere(27). Percentage cell proliferation inhibition was calculated and GI_50_ was presented with GraphPad Prism 6.0 software. 

***Flow cytometry***

SiHa and CaSki cells at a concentration of 1X 10^5^/ml/ well, were seeded in a 24-well plate. Cells were treated with respective near-GI_50_ doses along with DMSO controls and incubation for 24 hr. Cells treated with DMSO alone served as controls. The cells were washed twice with sterile PBS and then suspended in an HBSS permeabilization buffer for further processing. Three μg/ml FITC-coated anti-HPV-16 E6 antibody or 5 μg/ml FITC-coated anti-p53 antibody was added to the cells and further incubated for 30 min in the dark. After a couple of washes with ice-cold PBS, the cells were resuspended in the HBSS buffer and ten thousand events were acquired in a Guava easyCyte™ flow cytometer. The data were analyzed with InCyte Software from Millipore, (Burlington, CA USA) and respective percentage positive cells were presented with respect to their controls.

***Apoptosis detection assay***

SiHa and CaSki cells were treated with respective near-GI_50_ doses along with DMSO controls and incubated for 48 hr. Apoptosis was analyzed by using an Annexin V detection kit as described elsewhere (28). 

## Results

***High throughput virtual screening of ChemBridge against HPV-16 E6***

**Figure 1:**
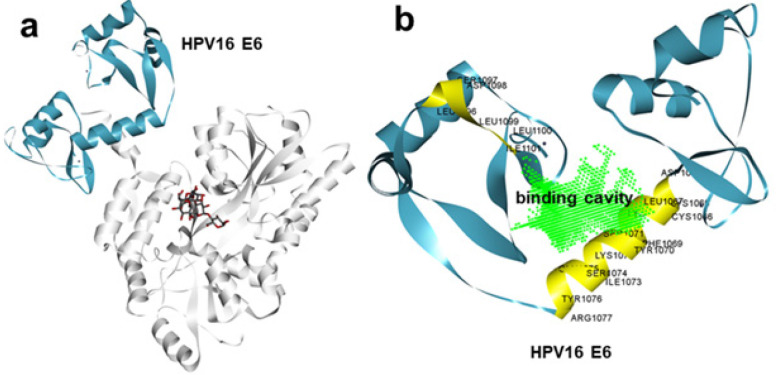
(a) Crystal structure of HPV-16 E6 (cyan) protein complexed with IRF receptor, (b) receptor cavity prediction analysis depicting predicted ligand binding cavity at the protein-protein interface of HPV-16 E6. Critical amino acids are highlighted in yellow color

**Figure 2:**
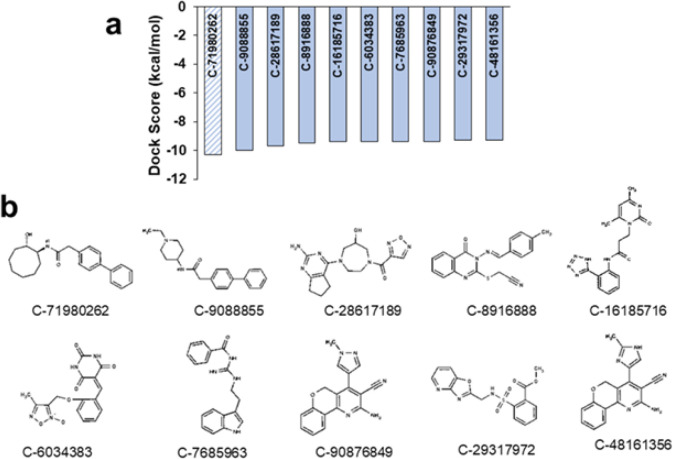
(a) Predicted top 10 compounds from ChemBridge library based on high-throughput virtual screening, ranked based on docking scores, (b) 2D representation of the top 10 molecules

Toward identifying a potent lead candidate, the experimental structure of HPV-16 E6 (6SIV) in complex with IRF3 was retrieved from the Protein Data Bank (PDB). The interaction region between HPV-16 E6 and the IRF3 LXXL motif was identified (Figure 1a). Key amino acid residues that were involved in the protein-protein interaction between HPV-16 E6 and the IRF3 region were highlighted (Figure 1b). Using the Discovery Studio visualizer, a ligand binding cavity within HPV-16 E6 was identified. This cavity comprises key residues involved in the interactions with HPV-16 E6 and has the potential to be targeted by small molecules to inhibit its activity. The ChemBridge small molecule library, which contains approximately 1.5 million small molecules, was chosen for virtual screening. These molecules were believed to have favorable drug-likeness scores. A high-throughput virtual screen (HTVS) of the entire ChemBridge library was conducted to predict the best lead molecules. Given the small size of the predicted binding pocket in HPV-16 E6, the compounds from the ChemBridge library were limited to those with a molecular weight between 150 to 350 kDa. The top compounds were extracted based on their docking scores, which are a measure of their predicted affinity for binding to HPV-16 E6 (Figure 2a). The 2D representation of the top 10 molecules is given in Figure 2b. Among the top compounds, compound C-71980262 was selected for further analysis (Figure 2). It showed a binding energy of -10.3 kcal/mol, indicating a strong potential for inhibiting HPV-16 E6. 

***Protein-ligand interaction profiling of C-71980262***

**Figure 3:**
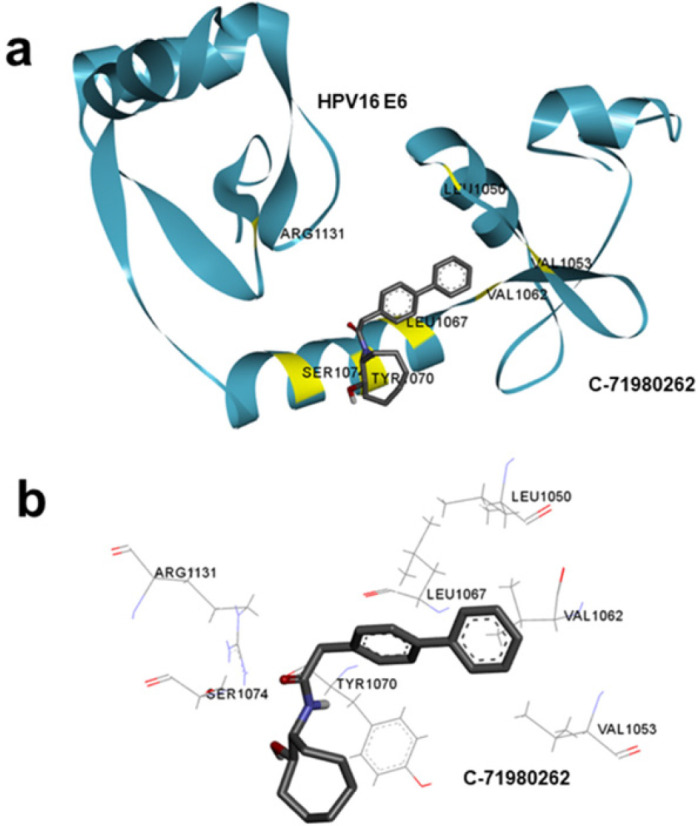
**.** (a) predicted binding pose for the top-ranked compound, C-71980262, depicting the binding mode with HPV-16 E6 protein. Interacting amino acids are represented and highlighted in yellow, (b) Two-dimensional representation of protein-ligand interaction analysis, showing C-71980262 and the interacting amino acids of HPV-16 E6

The compound C-71980262 identified by HTVS had a promising binding energy for HPV-16 E6. A favorable binding energy suggests strong interactions between the ligand (C-71980262) and the protein (HPV-16 E6), indicating a potentially effective binding between the two (Figure 3a). Protein-ligand interaction profiler analysis (PLIP) showed C-71980262 to bind at the hot-spot region in HPV-16 E6. The identified small molecule interacted with key amino acid residues on HPV-16 E6, including Arg1131, Ser1074, Tyr1070, and Val1053 (Figure 3b). These amino acids are crucial for protein functionality as well as its interaction with other proteins. Targeting these specific residues suggested that C-71980262 may disrupt the activity of HPV-16 E6 effectively. Therefore, C-71980262 was selected for further analysis.

***Atomistic molecular dynamic simulation of C-71980262 bound HPV-16 E6***

**Figure 4:**
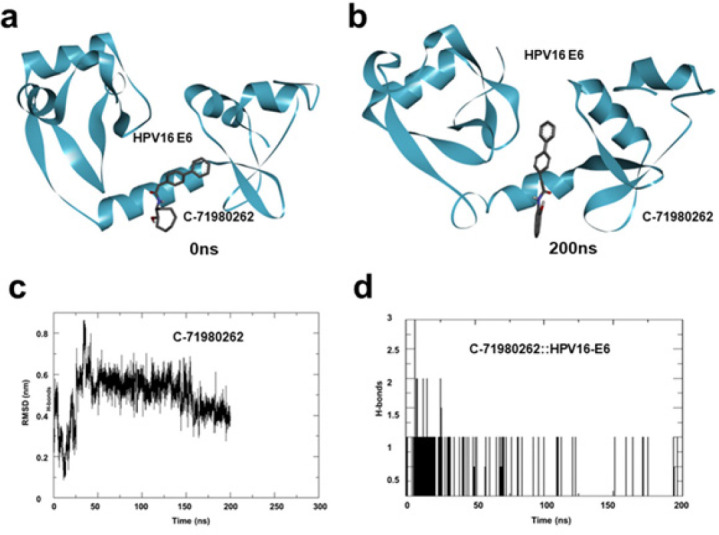
(a) Snapshots of simulation trajectories taken before and after the 200 ns simulation, showing the stability of the HPV-16 E6::C-71980262 complex, (b) Ligand Root Mean Square Deviation (RMSD), calculated from 200 ns simulation trajectory, (c) Number of H-bonds between C-71980262 and HPV-16 E6 calculated from a 200 ns simulation trajectory

**Figure 5:**
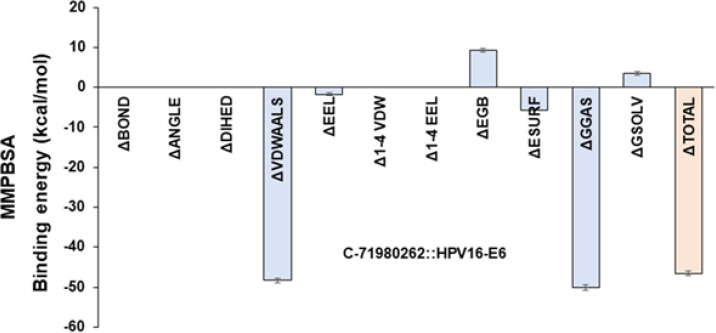
Predicted Gibbs binding free estimate of the lead compound C-71980262 to HPV-16 E6

In order to assess the binding stability and the dynamics of C-71980262 in complex with HPV-16 E6, a fully-solvated atomistic molecular dynamic (MD) simulation of HPV-16 E6::C-71980262 complex was performed for 200 ns. The complex was placed in a triclinic simulation box and fully solvated with Simple Point Charge (SPC) water. Additionally, counterions (NaCl) were added to mimic physiological salt concentration (0.15M NaCl). Prior to the simulation, the system was energy minimized for 5000 steps using the steepest descent method. This step helps in removing any steric clashes and prepares the system for equilibration. The simulation system was equilibrated for 300 picoseconds, which helped the system reach a stable state before starting the main simulation run. MD simulation was conducted for 200 nanoseconds using the GROMACS simulation software through a web-based application (www.sibiolead.com). During this time, the system was allowed to evolve and simulate the dynamics of C-71980262 bound to HPV-16 E6. The simulation snapshots taken before and after the 200 ns simulation were compared. This visual analysis indicates that C-71980262 remains stably bound to HPV-16 E6 throughout the simulation (Figure 4a, b). The Root Mean Square Deviation (RMSD) of the ligand (C-71980262) was calculated throughout the simulation. The RMSD value of less than 0.2 nm suggested a stable binding of C-71980262 to HPV-16 E6 (Figure 4c). Hydrogen bond (H-bond) analysis was performed to assess the stability of the interactions between C-71980262 and HPV-16 E6. The results show a stable H-bond pattern for C-71980262 throughout the simulation, indicating that the ligand maintains consistent interactions with the receptor (Figure 4d). Furthermore, toward identifying the binding stability of C-71980262, MMPBSA (Molecular Mechanics Poisson Boltzmann) based binding energy was estimated. For this, 50 frames comprising the 200 ns simulation were used. Binding free energy was estimated using the gmx_MMPBSA tool. Results show a binding energy estimate of -46.54 kcal/mol for C-71980262, which indicates a favorable and robust binding (Figure 5). Collectively, these MD simulation results suggested that C-71980262 could bind avidly and stably to HPV-16 E6. 

***C-71980262 inhibited HPV-16 positive cervical cancer cell proliferation***

**Figure 6:**
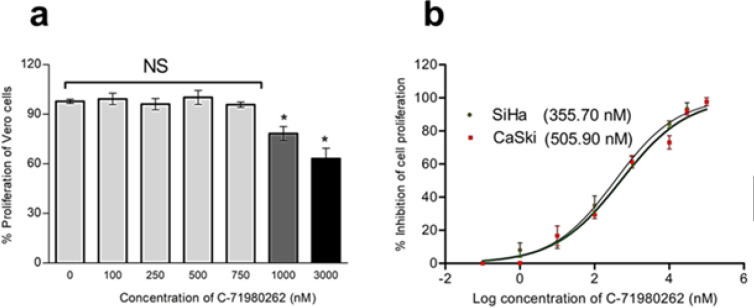
(a) Effect of C-71980262 on proliferation of Vero cells. The compound showed no toxic effect on Vero proliferation up to 750 nM concentration. Results are from three individual experiments expressed as mean±SD and statistically significant at *P≤0.05, (b) GI50 values of C-71980262 on proliferation of SiHa and CaSki cells. Results were representative of three individual experiments performed in triplicate

Prior to checking the effects of the compound on the cancer cells, the maximum tolerated dose (non-toxic concentrations) of the compound in the non-cancerous Vero cells was determined. No change in cell viability of Vero cells was found up to 750 nM of C-71980262 (Figure 6a). However, reduction in percentage cell viability was observed from 1000 nM concentration (Figure 6a). Dose-dependent decrease in the cell viability of both SiHa and CaSki cells was noted with C-71980262 treatments. The compound inhibited the SiHa cell proliferation with a GI_50_ value of 355.70 nM and CaSki cell proliferation with a GI_50_ value of 505.90 nM. (Figure 6b). The near GI_50_ concentrations, i.e., 350 nM for SiHa cells and 500 nM for CaSki cells, which were not toxic to the Vero cells were used for further evaluations. 

***C-71980262 inhibited HPV-16 -E6 and increased p53 in cervical cancer cells***

**Figure 7:**
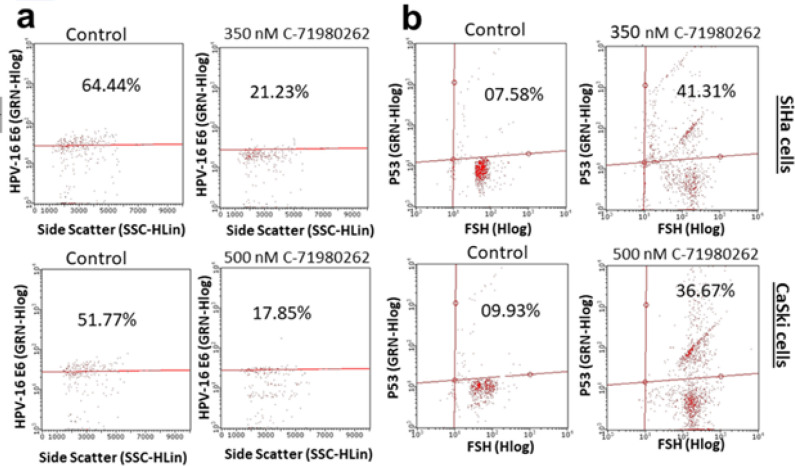
(a) Flow cytometric analysis for the effect C-71980262 on HPV-16 E6 percentage positive populations in cervical cancer cells. The compound reduced the HPV-16 E6 positive cell populations in SiHa and CaSki cells, (b) Effect of the compound on p53 positive population in SiHa and CaSki cells. C-71980262 up-regulated p53 in both these cells as evidenced by the increase in the p53 populations of both cervical cancer cells after C-71980262 treatment. Representative histograms from several repeats of the experiment are shown. Numerical values are expressed as mean±SD from three individual experiments

DMSO-treated SiHa and CaSki had 64.44% and 51.77% positive populations of HPV-16 E6 respectively (Figure 7a). Treatment of C-71980262 reduced the HPV-16 E6 positive papulation to 21.23% and 17.85% in SiHa cells and CaSki cells, respectively (Figure 7a). On the other hand, C-71980262 increased the percentage p53 cell population from 7.58% to 41.31% in SiHa cells and 9.93% to 36.67% in CaSki cells when compared to respective controls (Figure 7b). 

***C-71980262 induced early, late phase apoptosis in cervical cancer cells***

**Figure 8:**
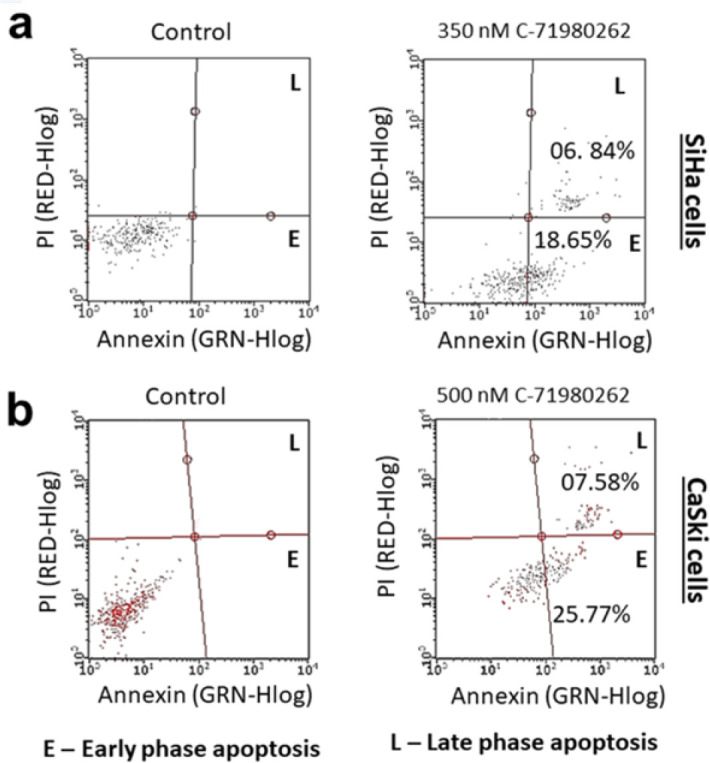
Flow cytometric enumeration for the early and late phase apoptotic cells by Annexin V staining in (a) SiHa and (b) CaSki cells after C-71980262 treatment for 48 hr. Representative histograms from several repeats of the experiment are shown. Numerical values are expressed as mean±SD from three individual experiments

Figure 8 represents the Annexin V assay by flow cytometry. This was performed by a kit method using Annexin V FITC reagent (X axis) and the Propidium iodide reagent (Y axis). The upper left quadrant with PI-positive cells is considered dead/ debris. Cells in the lower right quadrant that are positive for only Annexin V are considered early apoptotic cells. Cells in the top right quadrant that are positive for both Annexin and PI are considered late-phase apoptosis cells. The gating was performed in the control considering the maximum cluster of healthy cells in the left bottom quadrant as seen in the controls of Figure 8. Treatment of 350 nM C-71980262 to SiHa cells induced 18.65 % early and 6.84 % late apoptotic cell populations (Figure 8a). CaSki cells, when treated with 500 nM C-71980262 showed 25.77 % and 7.58 % early and late apoptotic cell populations, respectively (Figure 8b). 

## Discussion

HPV is one of the major factors involved in the onset of cervical cancer. The serotype of HPV-16 is classified as a high-risk (HR) virus with its E6 protein playing a pivotal role in carcinogenicity. Given the high prevalence of HR HPV strains, like type 16 in the population and their association with cancer, drug development efforts aimed at targeting HPV-related oncogenic pathways, such as inhibiting E6-mediated p53 degradation, are considered of great importance (29). These efforts may lead to the development of therapeutics that can help prevent or treat HPV-related cancers, especially cervical cancer. To achieve this therapeutic outcome, it is essential to target E6 effectively.

The pursuit of identifying a potent lead candidate for inhibiting HPV-16 E6 is a multi-step and multifaceted process that involves structural analysis, virtual screening, and candidate selection. Using the diversity-based virtual screening process by Autodock-vina, ~850,000 compounds were virtually screened from the ChemBridge database. The present study faced certain challenges due to the limited availability of complete E6 protein crystal structures and the absence of reported co-crystallization of small molecule compounds with E6. These factors contribute to the inherent risks associated with virtual screening in this particular research. Given these limitations, the study had to rely on screening candidate compounds based on the structural characteristics of HPV-16 E6 and available data. While this approach may pose certain challenges and uncertainties, it underscores the importance of leveraging the information at hand and employing computational techniques to identify potential drug candidates that can interact with E6 effectively.

Previous structural studies of E6 have revealed the existence of a hydrophobic site that plays a critical role in its interactions with p53 and E6AP (30-32). Targeting the hydrophobic site in E6 can prevent the formation of heterodimer complexes with p53 and E6AP which offers a framework for the design of targeted drugs. By inhibiting these interactions, it becomes possible to halt the process of oncogenesis (33). This requires a comprehensive understanding of the druggable pockets within E6 and their interactions with various protein molecules. 

According to the docking scores, the top 10 molecules were found to have a high affinity for HPV-16 E6. PLIP analysis showed these molecules bind to HPV-16 E6 and can fully occupy the binding sites of E6. The binding stability of C-71980262 was analyzed using MD simulation. The simulation snapshots taken before and after the 200 ns simulation were compared, revealing that C-71980262 maintained a stable binding to HPV-16 E6 throughout the simulation. This visual analysis underscores the robust interaction between the ligand and the protein. Additionally, H-bond analysis indicated the stability of interactions between C-71980262 and HPV-16 E6. The results demonstrated a consistent H-bond pattern for C-71980262, further emphasizing its steady interactions with the receptor. Overall, the MD simulation results suggested that C-71980262 exhibits avid and stable binding to HPV-16 E6. It is noteworthy that the identified compound C-71980262 binds at the hydrophobic site on E6, which is an indicator of the potential for C-71980262 to inhibit downstream activities of HPV-16 E6 that could have important implications in the context of HPV-related diseases, such as cervical cancer. The remarkable binding stability and energy estimate highlight the therapeutic potential of C-71980262 in combating HPV-related conditions.

Treatment of C-71980262 inhibited the growth of cervical cancer cells significantly, indicating that targeting E6 alone is beneficial for controlling cell proliferation in these cells. Moreover, the effective drug concentration for inhibiting cervical cancer cell proliferation was well below the non-toxic dose of the compound. The E6 protein mediates the ubiquitination and degradation of p53 in combination with E6AP (34). The role of p53 as a tumor suppressor is well-established, as it regulates cell cycle checkpoints, DNA repair, and apoptosis (35). When p53 is degraded, it ceases to discharge its tumor-suppressing functions effectively, which leads to tumorigenesis (36). Therefore, formulating drugs or therapeutic interventions that can disrupt the HPV-16 E6-E6AP-p53 interaction or stabilize p53 function could be a valuable approach in the fight against HR-HPV-associated cancers (37, 38). Both the extrinsic and intrinsic pathways play a crucial role in maintaining a balance between pro-apoptotic and anti-apoptotic proteins (39). However, the presence of oncogenes can disrupt this delicate equilibrium. When the prominent oncogene E6 is effectively silenced using small molecules, it leads to an increase in p53 levels (21). This elevation in p53 levels triggers the activation of PUMA (p53 up-regulated modulator of apoptosis), which, in turn, promotes and guides the cell toward apoptosis (40). The observed effects of C-71980262 in inhibiting HPV-E6 and up-regulating p53 to induce early and late phase apoptosis in SiHa and CaSki cells were on par with the aforementioned studies, thereby indicating the efficacy of the lead compound against HPV-16 positive cervical cancer cells. 

## Conclusion

Compound C-71980262 was identified to bind HPV-16 E6 as a result of computational evaluations like virtual screening and molecular dynamic simulations. The compound inhibited the proliferation of HPV-16-positive SiHa and CaSki cells. While E6 was positive, SiHa and CaSki cell populations were reduced upon C-71980262 treatment, and the compound increased p53 positive populations in these cells to promote early and late phase apoptosis. The outcomes of this research could potentially lead to the development of an effective anticancer agent against HPV-linked cervical cancers. However, it is recommended to conduct further evaluations of C-71980262 in both *in vitro* and *in vivo* models to solidify its status as a leading drug candidate for inhibiting HPV-16. 

## Data Availability

All data used in this manuscript are available from the author and can be provided upon reasonable request for noncommercial purposes.
